# Effects of ambient wind on droplet deposition uniformity in orchard air-assisted sprayers

**DOI:** 10.1038/s41598-025-24418-5

**Published:** 2026-01-16

**Authors:** Tao Xu, Xue Li, Longpeng Ding, Yannan Qi, Haocheng Lu, Wen Xiao, Xiaolan Lv, Jingbin Li

**Affiliations:** 1 Xinjiang Production and Construction Corps Key Laboratory of Modern Agricultural Machinery, Shihezi, 832003 China; 2 Institute of Agricultural Facilities and Equipment, Jiangsu Academy of Agricultural Sciences, Nanjing, 210014 China; 3 School of Mechnical and Electrical Engineering, Shihezi University, Shihezi, 832003 China; 4 College of Engineering, Nanjing Agricultural University, Nanjing, 210095 China; 5 Huaiyin Institute of Technology, Huaian, 223003 China

**Keywords:** Droplet deposition, Ambient wind, Wind speed, Spraying conditions, Air-assisted sprayer, Engineering, Environmental sciences

## Abstract

To investigate the influence of the ambient wind on pesticide droplet deposition and drift loss patterns inside and outside the canopy of fruit trees, spraying tests were carried out under different ambient wind conditions. The results showed that due to the shielding effect of branches and leaves, droplets mainly concentrated in the middle and lower part of the canopy, and the coverage rate of droplets in the middle and lower canopy was 27.14% and 39.46% at a wind speed of 0.47 m/s. The droplet coverage rate decreased to 17.88% in the upper canopy, and the coefficient variation increased to 118.65. In addition, upwind spraying increased ground deposition of droplets in the downwind of 0–4 m and the airborne drift of droplets at a height of 3–4.5 m. Still, it decreases the deposition distance of droplets in the downwind. Compared with the direction of tractor moving against the wind, downwind driving reducing the penetration of droplets in the canopy, the loss of droplets in the air and the ground deposition in the downwind. The research and findings presented in this study provide critical insights for predicting droplet drift and ground deposition under varying wind conditions, optimizing field operational parameters, improving canopy distribution uniformity, and enhancing pesticide utilization efficiency.

## Introduction

The fruit industry is the third largest agricultural planting industry in China^1^. Preventing and controlling orchard pests and diseases is integral to fruit tree management^2,3^. At present, it mainly relies on chemical prevention. According to statistics, fruit trees must be sprayed 8–15 times during their annual growth cycle^4^. Nevertheless, the fruit tree canopies are tall and densely foliated, making it difficult for droplets to penetrate, so the Food and Agriculture Organization of the United Nations strongly recommends using air-assisted spraying technology^5^, which uses the axial airflow generated by the sprayer fan to carry pesticide droplets through the canopy and increase deposition^6,7^. The air-assisted sprayer is the primary pest and disease control model in orchards.

Domestic and foreign researchers have extensively researched the distribution of the sprayer’s airflow inside and outside the canopy^8,9^. For example, Lü et al.^10^ set a reasonable angle of the deflector to explore the change of the airflow distribution in the orchard to make the air distribution match the canopy of fruit trees and achieve the purpose of profiling spray. Ding et al.^11^ adopted the double fan superposition method to solve the asymmetry problem of the wind field on both sides. Godyn et al.^12^ arranged two axial fans up and down and adjusted the fans’ vertical distance to optimize the canopy’s airflow field distribution. Garciaramos et al.^13,14^ designed a sprayer with two reverse-rotating axial flow fans. Compared with a single fan, the airflow field generated by the fans during the spraying operation significantly increased the amount of droplets deposited to the target. The above studies realize the profiling spray operation by adjusting the distribution of the outflow field of the sprayer fan. However, the influence of external natural wind is mainly reflected in two aspects: the change in wind speed will cause the movement trajectory of spray droplets in the air to shift, which will further affect the deposition efficiency of spray droplets. In contrast, under strong wind conditions, droplets are likely to be lost, and too low wind will lead to uneven penetration of droplets, which will affect the uniform distribution of droplets in the canopy and reduce the effective utilization rate of pesticide^15,16^. It increases the application cost and may cause potential adverse effects on the surrounding ecological environment, such as the impact of non-target organisms and soil and water pollution^17^. Therefore, it is of great theoretical and practical significance to study the influence of ambient wind conditions on the droplet behavior of orchard air-assisted sprayers to optimize spraying technical parameters^18^, improve application effect, and reduce environmental risk.

A field study^19^ was conducted to determine wind effects on spray deposition at different distances from an air-assisted sprayer. The results showed that when the wind direction was perpendicular to the spray direction, the deposition of droplets increased at downwind. Perkins et al.^20^ studied the variability in resulting spray drift across downwind distances was assessed alongside wind speed measured at on-site weather stations. The study concludes that trials with relatively higher wind speeds were associated with more significant spray drift deposition at relatively close sampling distances downwind from the application area. Nordby et al.^21^ experimentally studied the droplet loss characteristics at different wind speeds and concluded that when wind speed increased from 1.5 to 4.0 m/s, spray drift increased from 1.4% to 2.9% and field spraying should not be carried out when the wind speed exceeds 3 m/s^22,23^. Duga et al.^24^ explored how ambient wind speed influenced airborne drift distribution of droplets by using CFD methodology. Simulation results showed that a wind direction opposite to the spraying direction, increased of the ground deposition and the amount of spray remaining in the air.

It is of great significance to understand the influence mechanism of wind on the distribution of pesticide droplets inside and outside the canopy of fruit trees to optimize the operating parameters of the air-assisted sprayer and improve the effective utilization rate of pesticides^24^. However, the current design and operation specifications of sprayers often need to be thoroughly considered, as changes in the wind conditions can result in poor results in practical applications^25^. This paper explores the distribution of droplets deposited in the canopy of fruit trees by ambient wind, and the distribution laws of droplets drift in the air and deposition on the ground to provide a scientific basis for optimizing spray operation and improve the effectiveness and safety of application of pesticides in orchards.

## Materials and methods

### Experiment environment

The field test was conducted in the open field of the Institute of Agricultural Facilities and Equipment of Jiangsu Academy of Agricultural Sciences. The outdoor temperature was 33.5–35.7℃, and the relative humidity was 70.2–72.6%. Before the test, the SP3000 three-dimensional anemometer was mounted upward in an open position for unobstructed airflow to monitor and record the wind speed and direction at 4 m above the ground in real time.

### Instruments

**Fig. 1:**
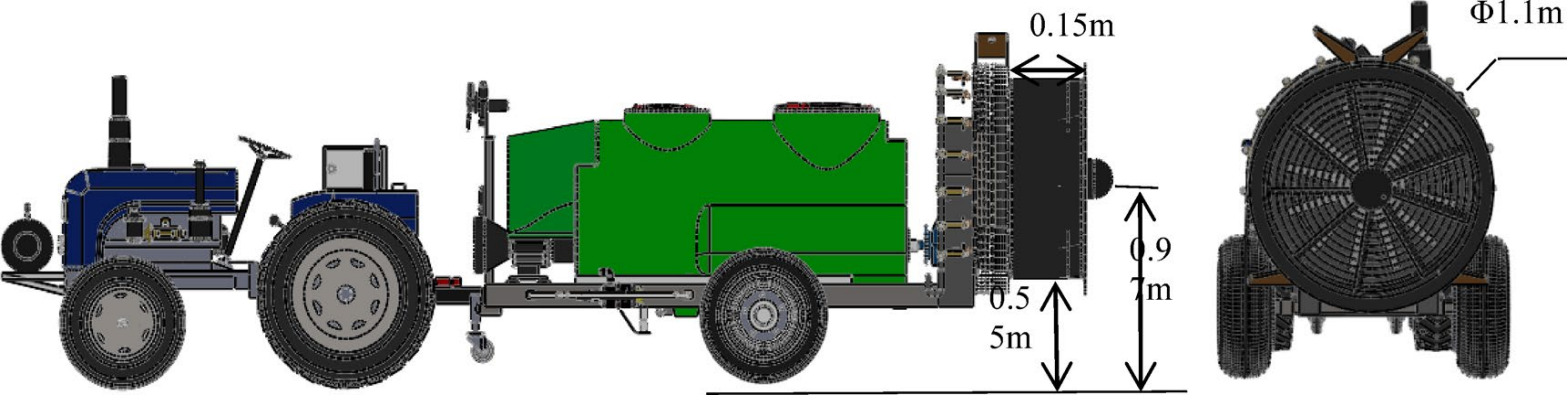
3WQF-1000 orchard air-assisted sprayer.

This experiment used a 3WQF-1000 traction-type orchard air-assisted sprayer developed by the Institute of Agricultural Facilities and Equipment of Jiangsu Academy of Agricultural Sciences, as shown in Fig. 1. The related technical parameters are shown in Table 1. The machine has the characteristics of a uniform and symmetrical wind field, profiling spraying, etc. The sprayer is suitable for large-scale standardized orchard.

**Table 1:** Technical parameters of 3WQF-1000 trailed Air-assisted Sprayer.

Index	Parameters
Hitch type	tractor-trailer
Matched power (KW)	36.8 with(50 horsepower)tractor
Chassis form	wheel type
Grade ability	≤10°
Turn radius(m)	≤ 4
Tank capacity(L)	1000
Type of the tank	special-shaped, no residual, high pressure washing system
Liquid pump	diaphragm pump 137(L/min)
Diameter of the fan(mm)	820(12 propeller blades)
Rotational speed of the fan(r/min)	1900 ~ 2300 (adjustable)
Blowing rate(m^3^/h)	1800 ~ 4500 (adjustable)
Spray range on one side(m)	≧ 10
Nozzle	Nozzle with anti-drip valve, HCC80°

### Field trials

#### Theoretical analysis

**Fig. 2:**
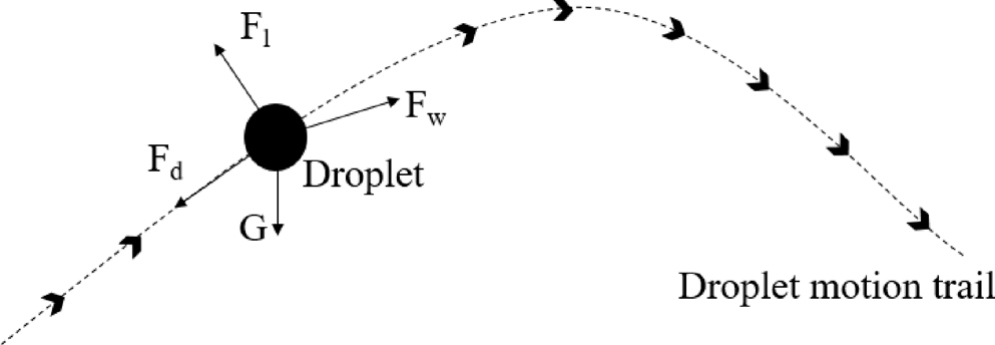
The force condition of a single droplet.

Liquid is atomized into droplets by a nozzle under hydraulic pressure and subsequently dispersed into the air and reach the target along the direction of the trajectory. Each droplet experiences several key forces: gravitational force (G), drag force (F_d_), lift force (F_l_), and the force exerted by the wind field (F_w_), as illustrated in Fig. 2. Drag force (F_d_), arising from the friction between the moving droplet and surrounding air molecules, acts opposite to the droplet’s velocity vector. Lift force (F_l_), generated by velocity gradients in the airflow around the droplet, acts perpendicular to its direction of motion. The wind field force (F_w_) is the combined force of turbin-induced wind and ambient wind. The greater F_w_, the farther the droplet travels. These forces expressed as Eqs. (1)- (4).

1 $${F_d}=\frac{1}{2} \cdot {C_d} \cdot \rho \cdot A \cdot {\nu _r}^{2}$$

2 $${F_l}=\frac{1}{2} \cdot {C_l} \cdot \rho \cdot A \cdot {\nu _r}^{2}$$

3 $${F_W}=\frac{1}{2} \cdot {C_W} \cdot \rho \cdot A \cdot {\nu _r}^{2}$$

4 $$G=\frac{4}{3} \cdot \pi \cdot {r^3} \cdot {\rho _d} \cdot g$$

Where, *F*_*d*_ is drag force, N; *C*_*d*_ is drag coefficient; *ρ* is air density, 1.225 kg/m³; *A* is windward area of the droplet, m^2^; *v*_*r*_ is relative movement speed of droplets, m/s; *F*_*l*_ is lift force, N; *C*_*l*_ is lift coefficient; *F*_*w*_ is wind force, N; *C*_*w*_ is wind coefficient; *r* is radius, m; *ρ*_*d*_ is droplet density, 1000 kg/m³; *g* is gravitational acceleration, 9.81 m/s².

During spraying, the tree canopy is modeled as a porous medium. When air and droplets through the canopy under elevated pressure and velocity conditions, the resultant directional changes in the airflow path inevitably lead to energy dissipation, thereby affecting the distribution of droplets in the canopy^26^. As airflow velocity increases, the surface pressure on the target progressively rises, generating a non-uniform pressure field within the flow domain. This pressure gradient induces droplet acceleration during transport, ultimately causing detachment from the target and subsequent drift. Conversely, insufficient airflow velocity (< 3 m/s) results in inadequate droplet kinetic energy for effective deposition, leading to escape phenomena^27^. 28. Nuyttens et al.^28^ found 90% of the emitted droplets deposited within 40 m distance downwind from the orchard at a wind speed of 4 m/s. At a wind speed of 2 m/s, the deposition within the 40 m distance from the orchard was more than 95%. Investigating the influence of wind on droplet deposition patterns within and outside the canopy during air-assisted spraying provides theoretical foundations and empirical data for optimizing operational parameters of air-assisted sprayers.

#### Operating condition

**Fig. 3:**
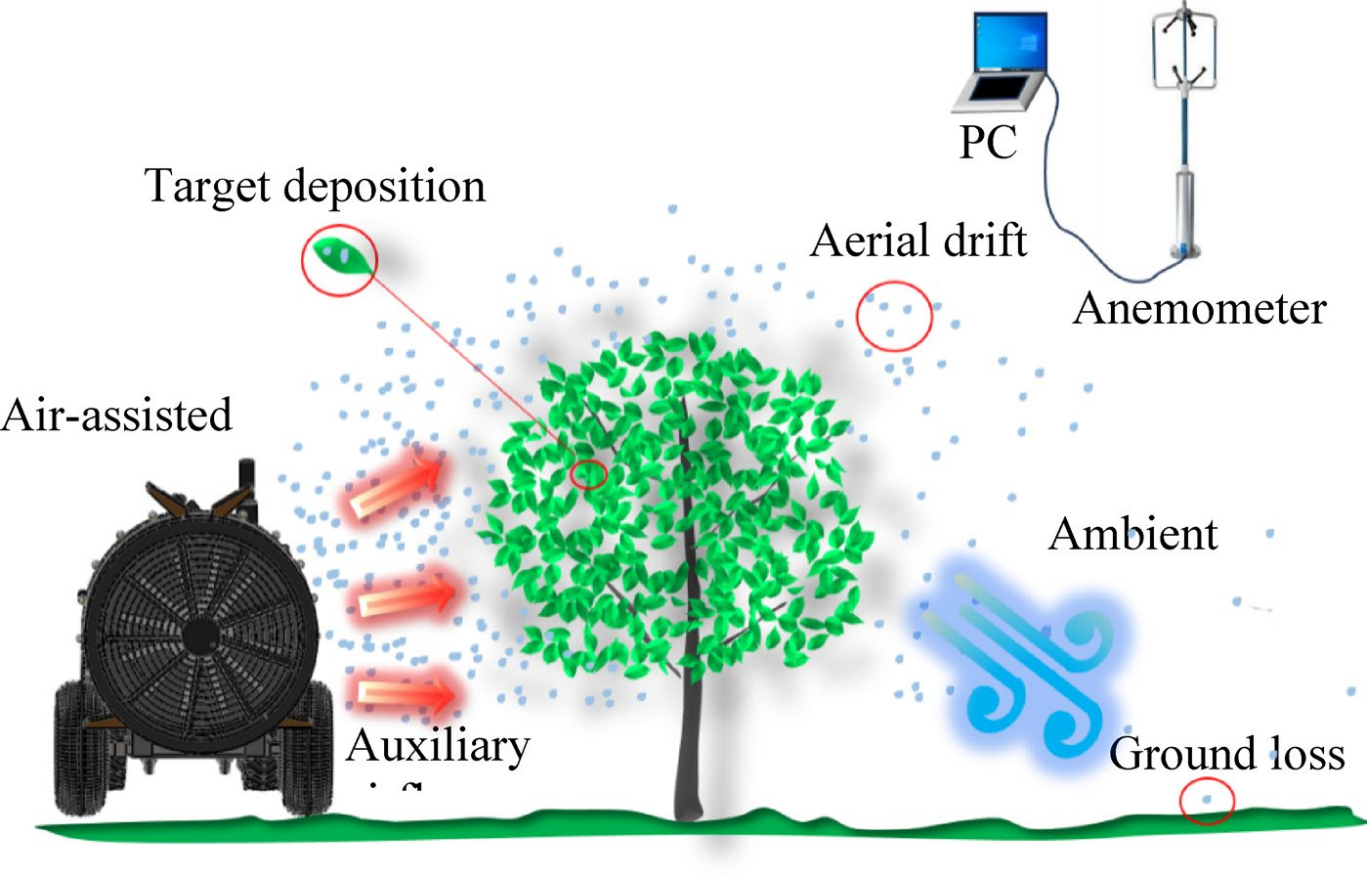
Field spraying.

During the test, the SP3000 three-dimensional anemometer monitored the wind speed, direction, temperature, humidity, and other parameters in real-time. The air-assisted sprayer moved at a constant speed of 0.48 m/s during the experiment, maintaining a horizontal distance of 2 m between its axis and the tree trunk, as illustrated in Fig. 3. The operational parameters of air-assisted sprayer included a fan rotational speed of 2000 rpm and an outlet wind velocity of 20 m/s^29,30^. The conical nozzle (HCC, 80°, ASJ) was used in the spray test. The droplet size is mainly distributed between 70 and 400 micrometers, so Brownian motion was not considered. The spraying system was configured with a working pressure of 0.4 MPa and a nozzle flow rate of 0.65 L/min. Six experimental groups were systematically tested using water-sensitive papers (Syngenta, dimensions 26 mm × 76 mm) to evaluate deposition patterns. Environmental conditions such as ambient temperature, humidity, and wind speed throughout the trials were recorded and documented in Table 2.

**Table 2:** Operating conditions of air-assisted sprayer.

Group	Temperature ℃	Relative humidity %	Wind speed m/s	Wind direction (True north is 0°, and clockwise is positive.	Spraying direction
A	35.5	72.2	0.47	West wind 254°	Upwind spraying; Upwind driving
B	35.6	72.4	1.04	South west wind 210°	Upwind spraying; Upwind driving
C	35.7	72.6	1.34	South wind 174°	Upwind driving
D	33.5	70.2	1.50	South wind 200°	Upwind driving
E	34.7	72.0	1.81	South wind 172°	Downwind driving
F	33.6	71.7	2.27	South wind 175°	Downwind driving

#### Sampling point arrangement

**Fig. 4:**
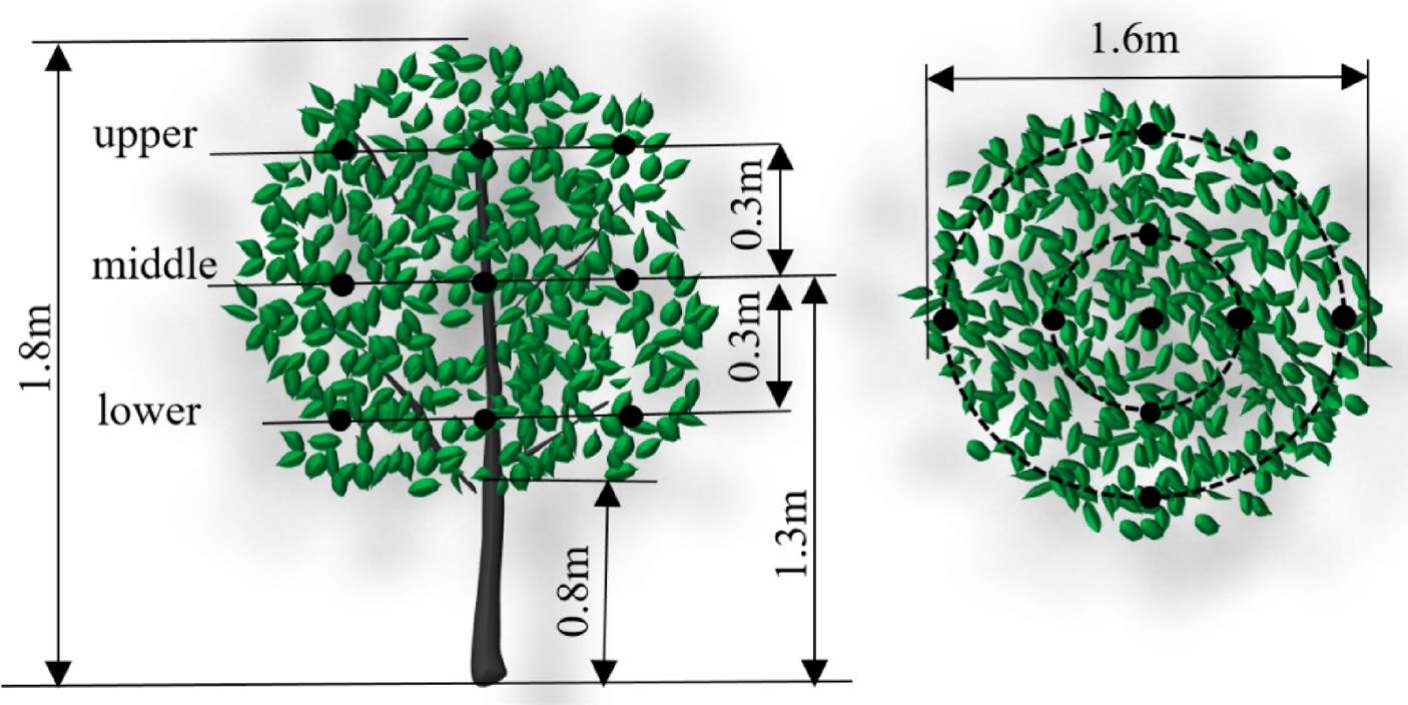
Test points in the canopy.

This study takes dwarf spindle-shaped pear trees as the research subject for artificial tree spray tests. The artificial tree was 1.8 m high, with a trunk height of 0.8 m, and a crown width of 1.6 m. The canopy leaf area index was 2.79 tested by the Plant Canopy Digital Image Analyzer (CI-110). According to the canopy morphology and stem density, the canopy was vertically divided into upper, middle, and lower parts with a vertical distance of 0.3 m. The distribution of points in the canopy is shown in Fig. 4. The distance between the adjacent two points is 0.35 m, and nine sampling points in each layer, totaling 27.

**Fig. 5:**
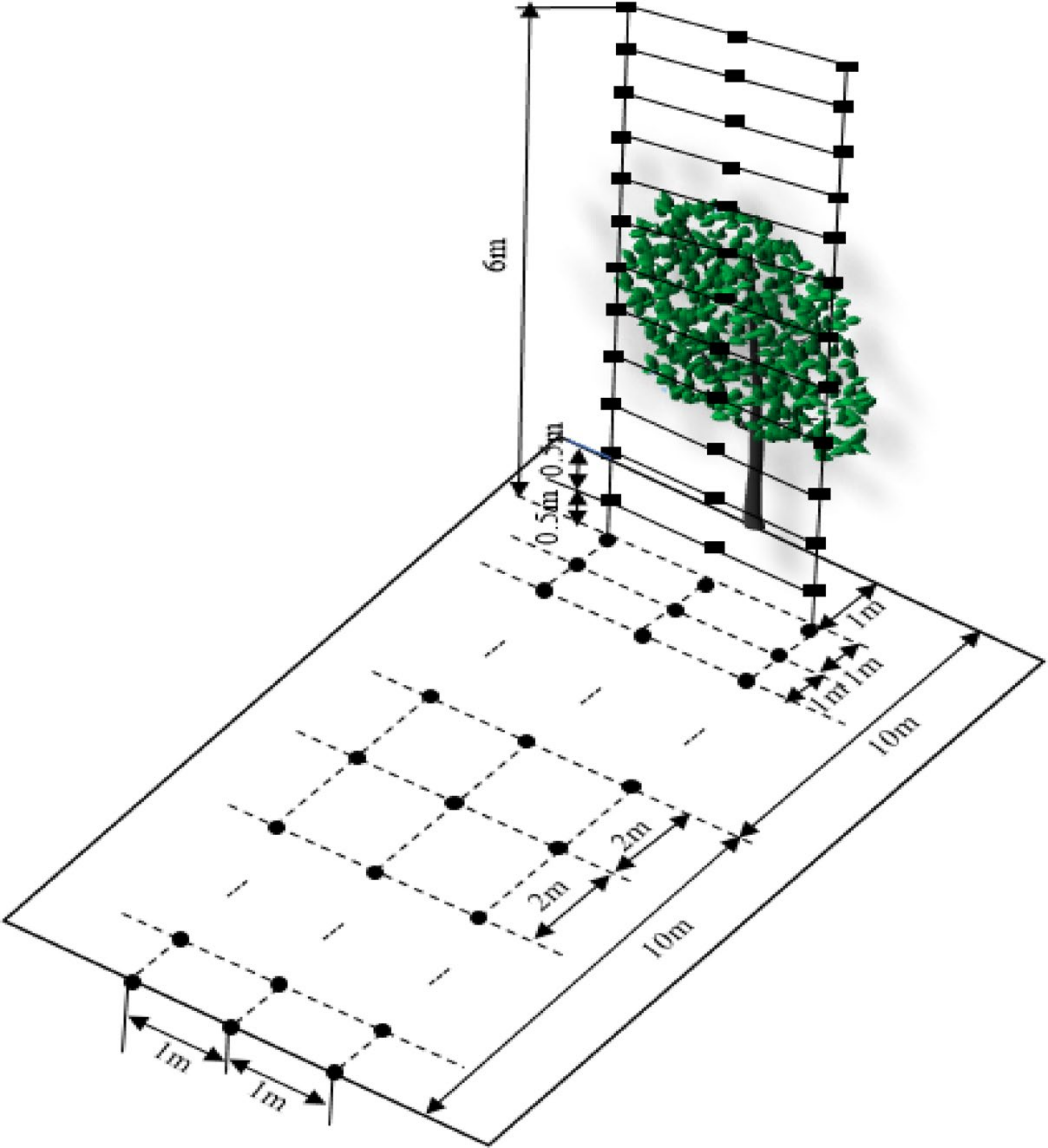
Test points for airborne drift and ground deposition.

The arrangement of sampling points for determining droplet ground deposition and drift is shown in Fig. 5. The horizontal distance of the droplet drift measurement device from the trunk was 1 m, the total height was 6 m, and the layer spacing was 0.5 m. Each layer had three sampling points, a horizontal spacing of 0.5 m, and totaling 36 sampling points. To determine the distribution of droplet ground deposition within 1–20 m from the tree along the spraying direction, three sampling points were arranged at intervals of 1 m and 2 m from the horizontal distance of 1–10 m and 10–20 m perpendicular to the sprayer path, respectively, with intervals of 0.5 m, totaling 45 sampling points.

#### Data collection and processing

After spraying, the water-sensitive paper in the canopy, air, and ground was promptly recovered and sealed with a paper envelope to prevent moisture. The water-sensitive paper was then returned to the lab and scanned into a JPEG image with 600 dpi pixels using an EPSON scanner. Then, Image J (National Institutes of Health, Bethesda), an image processing software, was used to measure the droplet coverage.

The coefficient of variation^31^ was adopted as the evaluation index to characterize the uniformity of droplet coverage rate at sampling points. The smaller the value, the more uniform the distribution of droplets at each sampling point. The formula for calculating the coefficient of variation is as follows:

4 $$CV=\frac{S}{{\bar {X}}} \times 100\%$$

5 $$S=\sqrt {\frac{{\sum\limits_{{i=1}}^{n} {{{\left( {{X_i} - \bar {X}} \right)}^2}} }}{{n - 1}}}$$

Where, *S* is the standard deviation of the sample collected in the experimental group. *X*_*i*_ is the index value of each sampling point. $\:\overline{X}$ is the average value of each sampling point. *n* indicates the number of sampling points.

## Conclusion

### Droplet coverage in the canopy

**Fig. 6:**
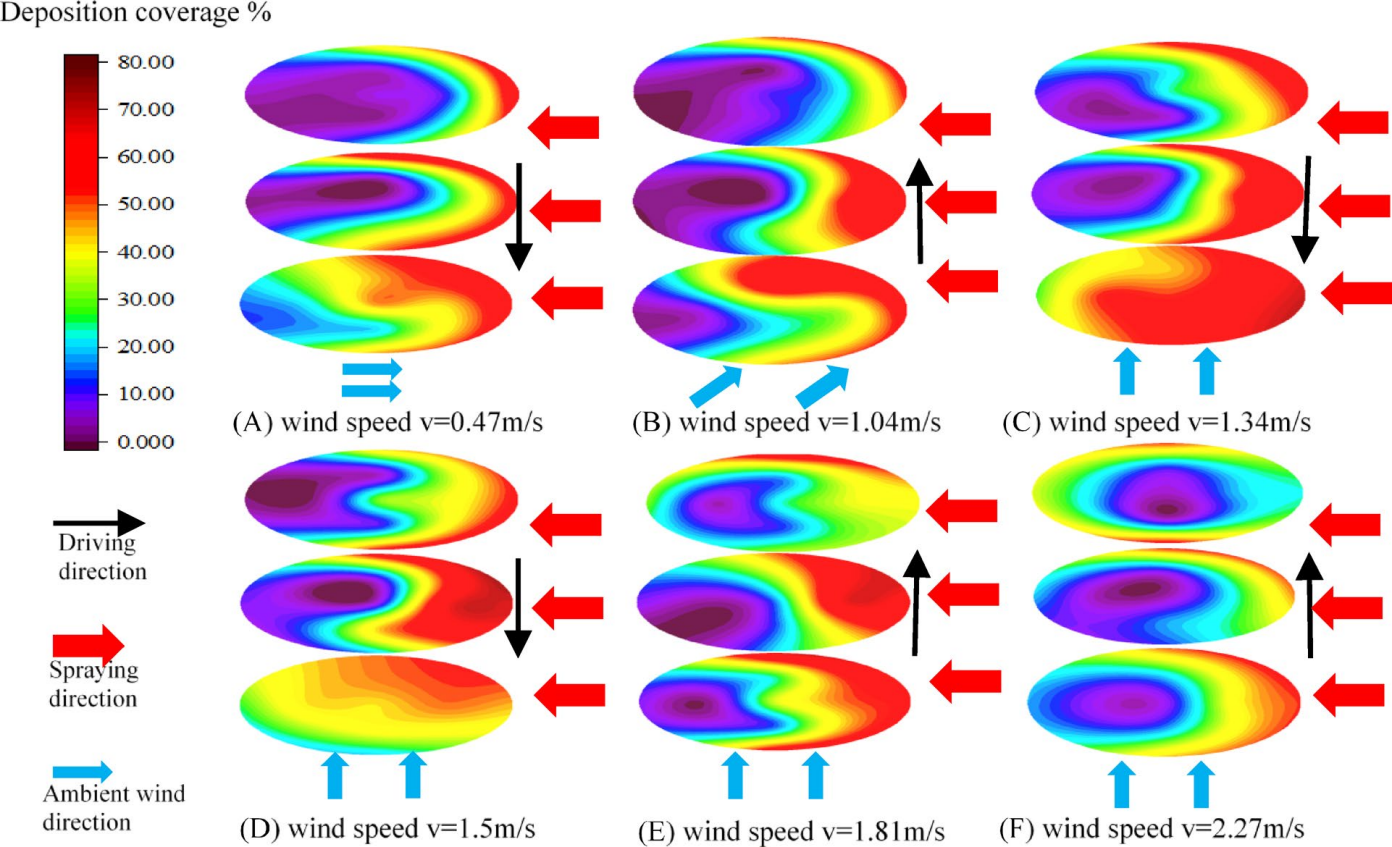
Deposition coverage of droplets in the canopy.

To explore the influence of ambient wind conditions on the deposition distribution of droplets in the canopy, spray tests were carried out on fruit trees with wind speed, wind direction, and driving directions as variables, and droplet deposition coverage at different positions in the canopy was statistically analyzed. With the tree trunk as the center, the direction of the sprayer is 0°, and the counterclockwise direction is positive. Plot the polar contour map of the coverage of droplets inside the canopy. Figure 6 shows the canopy’s upper, middle, and lower droplet deposition coverage under different wind conditions.

In general, due to the obstruction of airflow by the internal branches and leaves of the canopy, the ability of droplets to penetrate the canopy was weakened, resulting in droplets primarily deposited in the surrounding and lower of the canopy after reaching the canopy and the coverage rate of droplets was about 38%-50%. Due to the shielding effect of branches and leaves, the droplet deposition coverage in the middle of the canopy was the lowest, only about 3%. The results well agreed with previous literature^32^.

When the wind direction aligns with the direction of the sprayer’s movement, it is referred to as longitudinal wind. When it is perpendicular, it is called transverse wind. Based on the relative orientation of sprayer movement and droplet dispersion, the operation can be categorized into four distinct modes: driving downwind with downwind spraying, driving downwind with upwind spraying, driving upwind with downwind spraying, and driving upwind with upwind spraying, respectively. As shown in Fig. 6(A), the ambient wind speed was 0.47 m/s and upwind spraying. Compared with the wind speed at the fan outlet of 20 m/s, the airflow from the fan still played a leading role in the middle and lower of the canopy, droplets carried by airflow mainly concentrated in the lower part of the canopy and the edge of the middle part. The main reason is that the attenuation of the airflow within the canopy mainly occurs in the outermost layer of the canopy^33^. With the increase of vertical distance and the low leaf area index in the upper part of the canopy, the dominant role of the airflow generated by the fan was weakened, and the convection with the ambient wind was formed, which significantly weakened the penetration of droplets in the upper part of the canopy^19^. The average coverage rate of droplets in the canopy’s upper, middle, and lower parts was 17.88%, 27.14%, and 39.46%.

The wind, as shown in Fig. 6(B), was southwest wind. It could be divided into south wind and west wind. That is, there were both longitudinal wind and transverse wind. At this time, the spray condition was downwind driving and upwind spraying. It was observed that upwind spraying significantly reduced droplet penetration within the canopy while yielding poorer distribution uniformity. Figure 6(C) and Fig. 6(E) show the droplet deposition distribution within the canopy for the sprayer upwind driving and downwind driving, respectively, corresponding to wind speeds of 1.34 m/s and 1.81 m/s. It can be observed that upwind driving reduced lateral droplet drift and enhances droplet penetration. However, higher wind speeds reduced droplet penetration and deposition within the canopy^19^. Specific data can be found in Table 3.

The variation coefficient of coverage in the canopy was statistically analyzed, and the deposition stability was investigated. Although it is evident that wind condition such as speed and direction affects spray deposition in orchards, quantifying these effects is complicated^34^. This study treated wind conditions as a single variable when analyzing droplet coverage and the coefficient of variation. The results are shown in Table 3. Group A and B were upwind spraying, which weakens the penetration pattern of droplets and reduces the coverage rate in the canopy, resulting in uneven distribution of droplets. The coefficient of variation between group A and B were 81.36% and 86.58%, respectively. Group C and D were upwind driving, and group e and f were downwind driving. Compared with the downwind spraying, upwind spraying had a higher droplet deposition coverage rate, a smaller coefficient of variation between groups. Compared with group C, D and group E, F, the droplet deposition coverage rate in the canopy decreases and the coefficient variation increases as the wind speed increases.

**Table 3:** Statistics of droplets coverage in canopy.

Group	Position	Average coverage within the group %	Coefficient variation within the group %	Average covering density between groups %	Coefficient of variation between groups %
A	upper	17.88	118.65	28.16	81.36
	middle	27.14	91.93		
	lower	39.46	41.48		
B	upper	18.31	107.76	25.93	86.58
	middle	24.83	93.91		
	lower	34.64	60.80		
C	upper	30.58	69.81	38.62	58.56
	middle	32.38	77.64		
	lower	52.89	22.31		
D	upper	26.61	82.64	28.91	71.72
	middle	31.70	84.08		
	lower	28.43	31.99		
E	upper	28.61	59.15	31.55	68.38
	middle	30.79	74.89		
	lower	35.25	66.84		
F	upper	24.41	76.63	24.12	75.38
	middle	19.97	87.34		
	lower	27.99	62.71		

### Droplets drift in the air

**Fig. 7:**
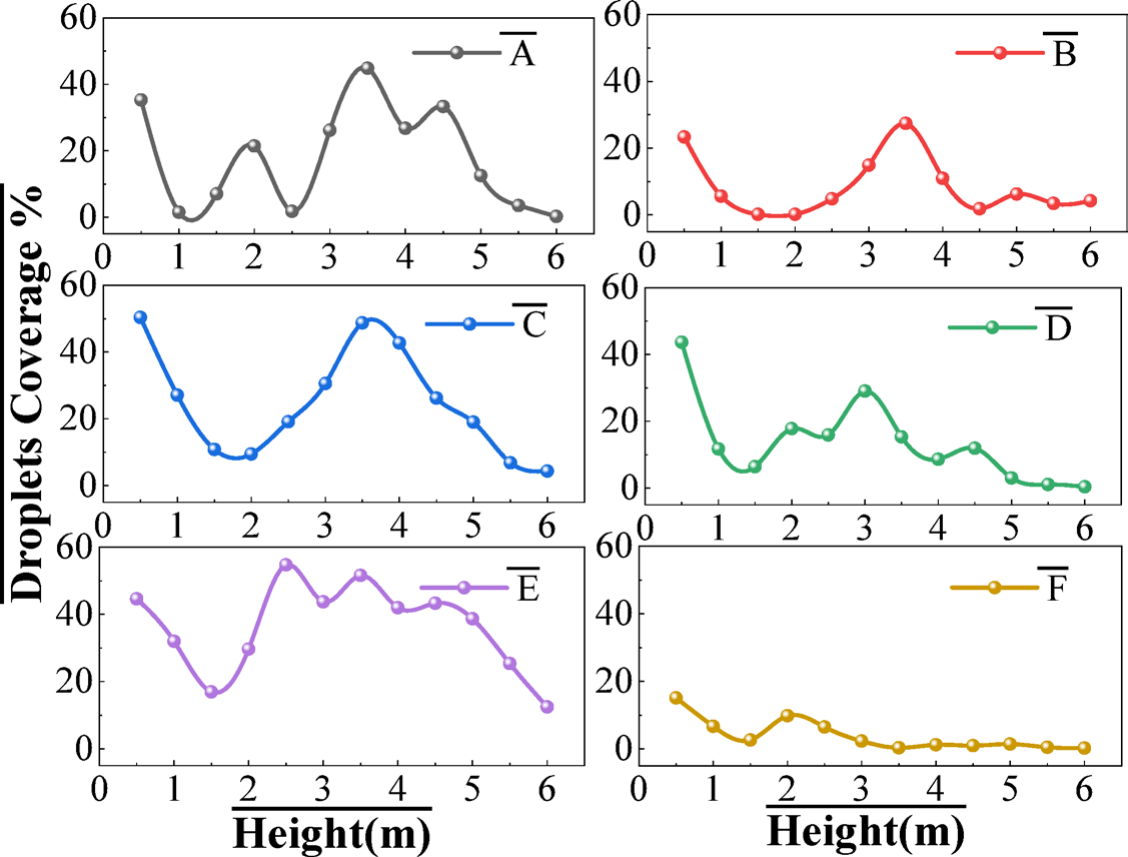
Droplet coverage of drift at different wind conditions.

Droplet airborne drift is a significant problem in orchard spraying, which causes pesticide waste and pollutes the environment^35^. The airborne loss of droplets under different wind conditions was tested to investigate the effect of wind on the droplets’ airborne drift. The statistical results of airborne drift coverage of droplets are shown in Fig. 7. It can be seen that under different wind conditions, the droplet deposition coverage rate in the air showed a decreasing trend at the height of 0.5–1.5 m. Duga et al.^24^ demonstrated that the capture efficiency of 20–50 μm droplets from apple tree canopies increased by 38–72%. Owing to the shielding effect of branches and foliage within the canopy, droplet penetration reached its minimum at 1.5 m height, resulting in troughs in the deposition coverage curve with coverage rates of approximately 2%–7%. Since then, with the increasing height, canopy porosity gradually decreased, and the shielding effect of branches and leaves was weakened. Therefore, under ambient wind and fan wind field, the airborne drift deposition coverage of droplets gradually increased, reaching the maximum at 3.5 m height. After that, the drift of droplets showed a fluctuating downward trend and eventually tended to 0 at 6 m height. In addition, the trend chart of droplet drift coverage changes gently during downwind spraying, and the airborne loss was significantly lower than that of other groups.

### Droplet deposition on the ground

**Fig. 8:**
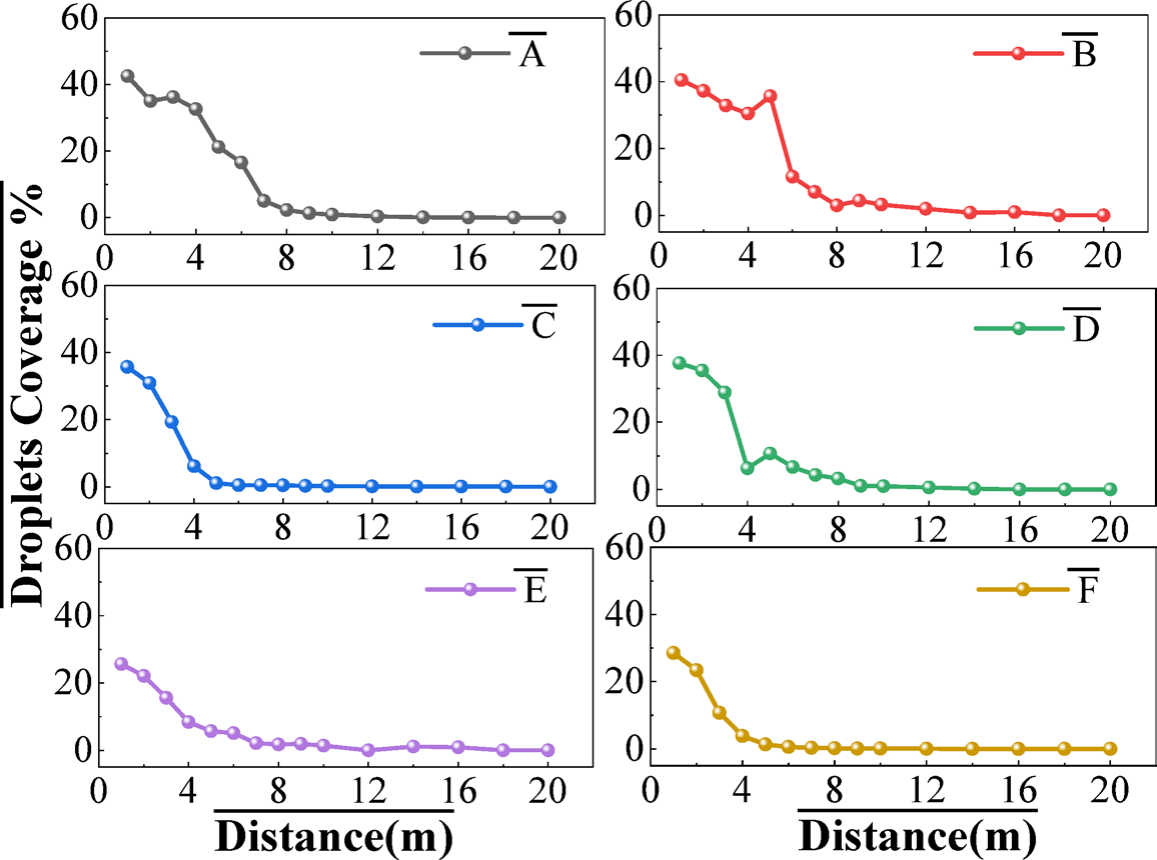
Droplets coverage on the ground at different wind conditions.

Vercruysse et al.^36^ illustrated that 39 and 29% of the amount of active ingredient sprayed are deposited on the ground in the orchard during the period when tree foliage was not fully developed and the full-leaf stage, respectively. Figure 8 shows the influence of wind speed on off-target deposition. It can be seen that the overall ground deposit coverage of droplets gradually decreases with the increase of distance, rapidly decreasing at 0–4 m and then decreasing to about 3% at 8–10 m and 0% at 16 m. Deposition decreased as the distance of the sampling location from the sprayer increased on both sides. Similar trends were reported by Zhu et al.^37^ and Derksen et al.^38^. Under the influence of transverse wind, the deposition rate of droplets in groups A and B at the horizontal distance of 0–4 m from the ground is significantly higher than in other groups. It can be concluded that upwind spraying reduces the ground loss of droplets. The longitudinal wind speeds of group e and group f were 1.18 m/s and 2.27 m/s, respectively. The higher the longitudinal wind speed, the lower the coverage rate of droplets along the spraying path. Al-Jumaili, et al.^39^ found that reducing the ground speed of the sprayer could increase the overall deposition.

## Discussion

Wind condition is one of the key factors affecting spray drift^40,41^. Understanding the impact of wind on spray effects is crucial for improving spray efficiency and reducing environmental pollution^42,43^. Under windless or light wind conditions (wind speed ≤ 1.5 m/s)^44^, droplets primarily move along their predetermined trajectory under the influence of gravity. However, as wind velocity increases, droplets are subjected to wind forces, causing them to deviate from their original trajectory^40^. Given the micron-scale dimensions of droplets, capturing individual droplet trajectories within the spray zone is challenging. This study therefore examined droplet distribution patterns within and outside plant canopies under different operational conditions, using single-spray-events droplet clouds as the research object. However, other critical spray parameters, such as sprayer air velocity, spray volume, spray distance, and travel speed^45–47^, are also significant factors influencing droplet deposition efficacy. Furthermore, existing studies predominantly focus on fruit trees with specific leaf area indices (LAI), making it difficult to generalize to fruit trees with varying LAI. To ensure research sustainability and comprehensiveness, future studies should investigate the droplet deposition distribution patterns by coupling multifactorial operational parameters across different LAI levels and fruit tree species. This will provide a data foundation for the adjustment of sprayer parameters under different wind speeds.

## Conclusions

Through the spraying test of fruit trees under different wind conditions, the deposition in the canopy and on the ground of droplets and airborne drift were analyzed. The influence of ambient wind speed and spray conditions on the penetration of droplets in the canopy was analyzed. The results indicated that the higher the wind speed, the lower the deposition coverage rate of droplets in the canopy along the spraying path, the greater the droplet drift loss in the air, and the smaller the droplet deposition amount on the ground. Under the influence of transverse wind, the deposition coverage rate of droplets increased at the horizontal distance of 0–4 m from the ground. It can be concluded that the upwind spraying substantially reduced the ground loss of droplets. These findings provide critical practical implications for optimizing sprayer parameters configurations in agricultural sprayer systems under varying natural wind regimes.

## Data Availability

Data will be made available by the corresponding author upon reasonable request.
